# Disruption of hippocampal synaptic transmission and long‐term potentiation by psychoactive synthetic cannabinoid ‘Spice’ compounds: comparison with Δ^9^‐tetrahydrocannabinol

**DOI:** 10.1111/adb.12334

**Published:** 2016-01-05

**Authors:** Alexander F. Hoffman, Matthew D. Lycas, Jakub R. Kaczmarzyk, Charles E. Spivak, Michael H. Baumann, Carl R. Lupica

**Affiliations:** ^1^ Electrophysiology Research Section, Cellular Neurobiology Branch National Institute on Drug Abuse Intramural Research Program Baltimore MD USA; ^2^ Designer Drug Research Unit National Institute on Drug Abuse Intramural Research Program Baltimore MD USA

**Keywords:** Drug abuse, electrophysiology, marijuana

## Abstract

There has been a marked increase in the availability of synthetic drugs designed to mimic the effects of marijuana. These cannabimimetic drugs, sold illicitly as ‘Spice’ and related products, are associated with serious medical complications in some users. *In vitro* studies suggest that synthetic cannabinoids in these preparations are potent agonists at central cannabinoid CB1 receptors (CB1Rs), but few investigations have delineated their cellular effects, particularly in comparison with the psychoactive component of marijuana, Δ^9^‐tetrahydrocannabinol (Δ^9^‐THC). We compared the ability of three widely abused synthetic cannabinoids and Δ^9^‐THC to alter glutamate release and long‐term potentiation in the mouse hippocampus. JWH‐018 was the most potent inhibitor of hippocampal synaptic transmission (EC_50_ ~15 nM), whereas its fluoropentyl derivative, AM2201, inhibited synaptic transmission with slightly lower potency (EC_50_ ~60 nM). The newer synthetic cannabinoid, XLR‐11, displayed much lower potency (EC_50_ ~900 nM) that was similar to Δ^9^‐THC (EC_50_ ~700 nM). The effects of all compounds occurred via activation of CB1Rs, as demonstrated by reversal with the selective antagonist/inverse agonist AM251 or the neutral CB1R antagonist PIMSR1. Moreover, AM2201 was without effect in the hippocampus of transgenic mice lacking the CB1R. Hippocampal slices exposed to either synthetic cannabinoids or Δ^9^‐THC exhibited significantly impaired long‐term potentiation (LTP). We find that, compared with Δ^9^‐THC, the first‐generation cannabinoids found in Spice preparations display higher potency, whereas a recent synthetic cannabinoid is roughly equipotent with Δ^9^‐THC. The disruption of synaptic function by these synthetic cannabinoids is likely to lead to profound impairments in cognitive and behavioral function.

## Introduction

**Figure 1:**
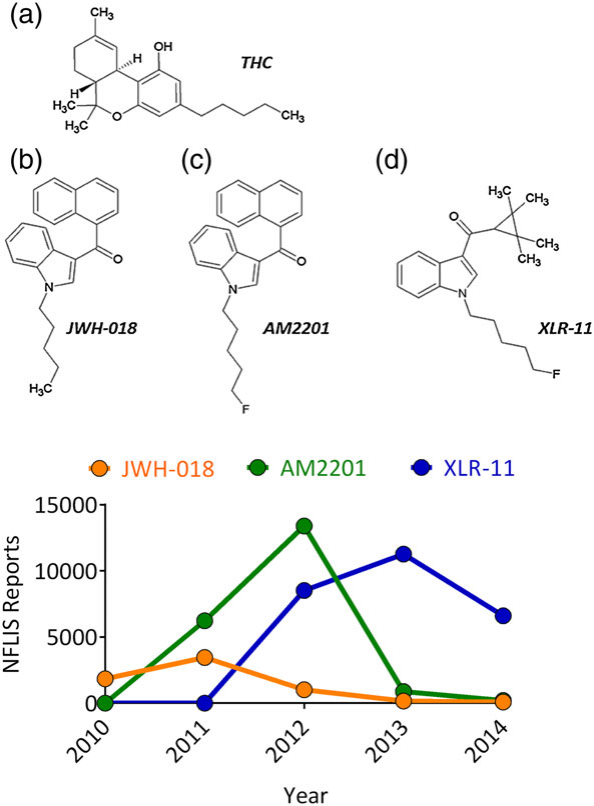
Chemical structure of the compounds tested in this study. (a) Δ^9^‐Tetrahydrocannabinol (THC), (b) JWH‐018, (c) AM2201 and (d) XLR‐11. Lower panel shows the total number of reports of the tested Spice compounds from the US National Forensic Laboratory Information System (NFLIS). Note the decline in reports of AM2201 and JWH‐018 and the marked rise in reports of XLR‐11 between 2012 and 2013. [Colour figure can be viewed at http://wileyonlinelibrary.com]

The potential therapeutic use of marijuana and related cannabinoids has led to a strong interest in developing compounds that can selectively target cannabinoid receptors but lack abuse liability (Izzo *et al*. 2009; Bisogno & Di Marzo 2010). Ironically, the development of such compounds by research laboratories across the world has provided clandestine chemists the framework from which to identify and synthesize potent drugs that mimic some of the psychoactive effects of Δ^9^‐tetrahydrocannabinol (Δ^9^‐THC). Consequently, there has been a global surge in the nonmedical use of synthetic cannabimimetic substances, marketed as ‘herbal incense’ and commonly known as ‘Spice’ (Logan *et al*. 2012; Lewin *et al*. 2014). Unlike the comparatively modest psychoactive and euphoric effects of marijuana, the use of Spice and related compounds has resulted in reports of severe anxiety, tachycardia, seizures and hallucinations (Schneir *et al*. 2011; Harris & Brown 2013). Nearly all of the identified chemical constituents of synthetic marijuana act as agonists at cannabinoid CB1 receptors (CB1Rs), and the psychoactive compounds in these preparations are frequently modified in response to legislative control imposed upon existing chemical structures (Vardakou *et al*. 2010; Seely *et al*. 2012). The structures of three of the most popular synthetic cannabinoids and their appearance in the National Forensic Laboratory Information System database are depicted in Fig. 1. The National Forensic Laboratory Information System data reflect the prevalence of particular synthetic cannabinoids that were confiscated by local, state and federal law enforcement. The naphthoylindole JWH‐018 (Fig. 1b) appeared frequently in Spice products confiscated during the years 2010 and 2011 in the United States but was rapidly supplanted by its fluoropentyl analog AM2201 (Fig. 1c; Seely *et al*. 2013). In the first half of 2013, the tetramethylcyclopropyl indole XLR‐11 (Fig. 1d) became much more prevalent than JWH‐018 or AM2201 (U.S. Drug Enforcement Administration Office of Diversion 2013), and this trend continued in 2014. Given the greater use of these synthetic marijuana preparations to avoid drug screen detection for Δ^9^‐THC, and increasing public health concerns regarding these compounds, there is a need to more fully characterize the neurobiological actions of these synthetic cannabinoids (Seely *et al*. 2012; Castaneto *et al*. 2014).

Among the best characterized effects of cannabinoids is the inhibition of neurotransmitter release from axon terminals via CB1R activation (Alger 2002; Hoffman & Lupica 2013). The cognitive deficits reported following acute or repeated use of marijuana (Abel 1970; Bolla *et al*. 2002) may reflect the effects of Δ^9^‐THC at CB1Rs located on both glutamatergic and GABAergic axon terminals in the hippocampus (Hájos *et al*. 2000; Sullivan 2000; Hoffman *et al*. 2007; Puighermanal *et al*. 2009). Most physiological studies have utilized synthetic agonists, such as WIN55212‐2 and CP55,940, to evaluate the central actions of cannabinoids (Hoffman & Lupica 2000; Robbe *et al*. 2001; Holderith *et al*. 2011), and relatively few studies have examined the effects of Δ^9^‐THC at central synapses *in vitro*, owing to its poor solubility and penetration into brain slices (Laaris *et al*. 2010; Hoffman & Lupica 2013). JWH‐018 has been evaluated in cultured hippocampal neurons (Atwood *et al*. 2010), and other Spice compounds have only recently been studied in pre‐clinical models (Atwood *et al*. 2011; Basavarajappa & Subbanna 2014). In this report, we directly compare the actions of JWH‐018, AM2201 and XLR‐11 with those induced by Δ^9^‐THC at hippocampal glutamatergic synapses.

## Methods

### Subjects

All studies utilized 4‐to 6 week‐old male wildtype C57BL6 mice or CB1^+/+^ and CB1^−/−^ mice bred on a C57BL6 background. Prior to initiating experimental work, studies were approved by the NIDA IRP Animal Care and Use Committee, in accordance with NIH guidelines.

### Hippocampal slice preparation

Mice were anesthetized with isoflurane and euthanized by decapitation. The brain was removed and placed in a beaker containing a modified ice‐cold artificial cerebrospinal fluid (aCSF) containing, in millimolar, *N*‐methyl‐d‐glucamine, 93; KCl, 2.5; NaH_2_PO_4_, 1.2; NaHCO_3_, 30; HEPES, 20; glucose, 25; sodium pyruvate, 3; MgCl_2_, 10; CaCl_2_, 0.5; and ascorbic acid, 5. Brain tissue was blocked and glued to the stage of a vibrating tissue slicer (Leica VT1200S, Leica Biosystems Nussloch, Germany) and submerged in modified aCSF. Transverse hippocampal slices (280 µm) were obtained and stored in standard aCSF containing, in millimolar, NaCl, 126; KCl, 3; CaCl_2_, 2.4; MgCl_2_, 1.5; NaH_2_PO_4_, 1.2; NaHCO_3_, 26; and glucose, 11. Slices were warmed in aCSF for 20–25 minutes at 35°C immediately after cutting and then allowed to gradually equilibrate to room temperature for at least 45 minutes prior to initiating recording. All solutions were oxygenated with 95% O_2_/5% CO_2_. We have previously found that endogenous adenosine can disrupt CB1R‐mediated inhibition of glutamate release in the hippocampus (Hoffman *et al*. 2010). Therefore, the selective adenosine A_1_ receptor antagonist, 8‐cyclopentyl‐1,3‐dipropylxanthine (DPCPX, 200 nM), was included in the aCSF throughout incubation and recordings. On average, three to four slices were used from a single animal per day. All drugs were tested in slices obtained from at least two subjects.

### Electrophysiological recording

Brain slices containing the hippocampus were submerged in aCSF in a slice chamber (RC‐26, Warner Instruments, Hamden, CT, USA) and continuously perfused with aCSF (2 ml/minute) using a peristaltic pump (Cole‐Parmer Instruments, Vernon Hills, IL, USA). Bath temperature was maintained at 30–32°C using an in‐line solution heater (TC324‐C and SH27‐B, Warner Instruments). Borosilicate glass electrodes (1.5 mm o.d. × 0.86 mm i.d., Sutter Instruments, Novato, CA, USA) were fabricated using a horizontal puller (P‐97, Sutter Instruments) and filled with aCSF. Electrodes were connected to the headstage of an AC amplifier (Model 1800, A‐M Systems, Sequim, WA, USA). Under stereomicroscopic visualization, a bipolar‐stimulating electrode consisting of twisted formvar‐insulated nichrome wire (50 µm; A‐M Systems) connected to a constant current stimulus isolation unit (DS3, Digitimer) was positioned in hippocampal area CA3 to activate Schaffer collateral fibers using 0.1‐millisecond pulses. The recording electrode was manually positioned in CA1 stratum radiatum using a micromanipulator and gradually lowered while monitoring the field excitatory postsynaptic potential (fEPSP) response. Once the optimal position was determined, stimulus intensity was varied to produce an input–output curve. Baseline responses were then set by adjusting the stimulus intensity to achieve 30–50% of the maximum response. In some experiments, paired pulse stimuli were delivered at an interval of 50 milliseconds. Stimulation, data acquisition and signal analyses were performed on‐line using an A/D board (PCIe‐6321, National Instruments, Austin, TX, USA) and WinLTP software (http://www.win-ltp.com; Anderson & Collingridge 2007). Responses were obtained every 30 seconds, and drugs were applied after obtaining at least 10 minutes of stable baseline recording. High‐frequency stimulation (HFS) consisted of three consecutive 100‐Hz, 1‐second trains, delivered 10 seconds apart. A three‐way valve was used to switch between control aCSF and drug‐containing aCSF. In most cases, drugs were applied for 40–60 minutes. To maintain consistent exposure times to cannabinoid agonists in long‐term potentiation (LTP) experiments, brain slices were pre‐treated for 90 minutes in the holding chamber (Collins *et al*. 1994, 1995). Thereafter, recordings were performed in either standard aCSF or aCSF containing the CB1 antagonist PIMSR1 ((5‐(4‐Chlorophenyl)‐1‐(2,4‐dichlorophenyl)‐4‐methyl‐3‐[(*E*)‐piperidinoiminomethyl]‐1*H*‐pyrazole)). The perfusion apparatus was washed with ethanol for 10–15 minutes between recordings.

### Chemicals and drug solutions

1‐Pentyl‐3‐(1‐naphthoyl)‐indole (JWH‐018), (1‐(5‐fluoropentyl)‐1*H*‐indol‐3‐yl)(naphthalen‐1‐yl)methanone (AM‐2201) and (1‐(5‐fluoropentyl)‐1*H*‐indol‐3‐yl)(2,2,3,3‐tetramethylcyclopropyl)methanone (XLR‐11) were supplied as dry powders by NIDA Drug Supply Program (Rockville, MD, USA). Stock solutions (1–10 mM) were prepared in dimethyl sulfoxide and frozen at −20°C until thawed for experiments. Δ^9^‐THC (200 mg/ml in EtOH) was provided through NIDA Drug Supply and was diluted to 10 mM in dimethyl sulfoxide. PIMSR1 was generously provided by Dr Herbert Seltzman (Research Triangle International, Research Triangle Park, NC, USA). AM251 and DPCPX were purchased from Tocris (Minneapolis, MN, USA). All drugs were dissolved to their final concentration in aCSF prior to each experiment. All other reagents were from Sigma‐Aldrich (St. Louis, MO, USA).

### Data analysis and statistics

Data are presented as mean ± standard error of the mean. Comparisons were made using *t*‐tests or ANOVA where appropriate, with a critical value for statistical significance set at *P* < 0.05. A Holm–Sidak's multiple comparisons test was used to measure the mean level of LTP between 55 and 60 minutes following HFS. Drug responses were defined as the change in the fEPSP slope at the time of the peak drug effect, typically 40 or 60 minutes after drug application. Prior experiments with cannabinoids have established that the relatively long drug onset time reflects partitioning of these highly lipophilic molecules into the brain slice. Responses were normalized to the baseline recording period. Dose–response curves were generated using a three‐parameter, global curve fitting nonlinear regression algorithm in Prism (GraphPad Scientific, San Diego, CA, USA):

Y=Bottom+Top−Bottom/1+10^LogEC50−X,

where Bottom and Top represent the plateaus and EC_50_ represents the agonist concentration that produces a response halfway between the top and bottom of the curve. The bottom was constrained to a value of 0. A global curve fit was first used to determine whether the top plateau differed among the family of curves (JWH‐018, AM2201, Δ^9^‐THC and XLR‐11). An additional sum of squares *F*‐test revealed that the maximum plateaus did not significantly differ (*F*_3,82_ = 1.203, *P* = 0.3139). In addition, a one‐way ANOVA revealed no significant differences in the maximal inhibition produced by each compound (*F*_3,17_ = 0.7656, *P* = 0.5289). Thus, the Top was allowed to be shared as a single value for all data sets, and the EC_50_ values were calculated.

## Results

### Inhibition of glutamate release by Δ^9^‐tetrahydrocannabinol

**Figure 2:**
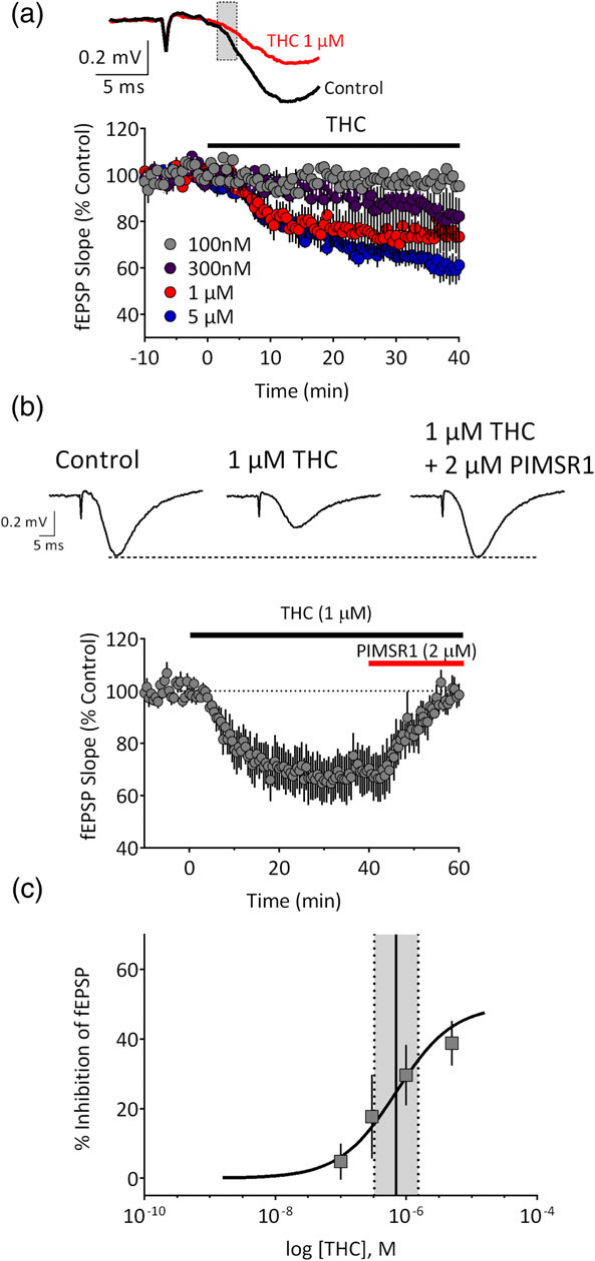
Concentration‐dependent inhibition of hippocampal glutamate release by Δ^9^‐tetrahydrocannabinol (THC). (a) Time course of the inhibition of the initial slope of fEPSPs (indicated by shaded box in upper traces) by Δ^9^‐THC across concentrations, applied at the time indicated by the horizontal bar. (b) Upper, representative averaged traces (*n* = 5–7 sweeps) demonstrating the effect of Δ^9^‐THC and reversal of its effects by the neutral CB1 antagonist PIMSR1. Lower, summary time course of recordings (*n* = 7 recordings) demonstrating the reversal of Δ^9^‐THC effects by PIMSR1. (c) Concentration–response curve for Δ^9^‐THC (*n* = 6–7 slices per concentration). The EC_50_ (solid line) was calculated to be 707 nM. Dashed lines indicate the 95% confidence interval. [Colour figure can be viewed at http://wileyonlinelibrary.com]

The cannabinoids found in synthetic marijuana preparations are presumably used as substitutes for the phytocannabinoid Δ^9^‐THC because they may mimic some of its pharmacological properties in the brain. Therefore, we first defined the effects of Δ^9^‐THC on excitatory synaptic transmission in the hippocampus *in vitro*. Glutamatergic fEPSPs, elicited by stimulation of Schaffer collateral axons, were recorded in area CA1 in transverse mouse hippocampal slices. Bath application of Δ^9^‐THC caused a concentration‐dependent reduction of fEPSP rising slope (Fig. 2a), and this was completely reversed by the neutral CB1 antagonist PIMSR1 (Hurst *et al*. 2006; Fig. 2b; *n* = 7, 99 ± 4% of control, *P* = 0.9534, paired two‐tailed *t*‐test). The maximal inhibition of fEPSPs was 39 ± 6% (*n* = 7), with an EC_50_ of 707 nM (95% CI = 333–1053 nM). These effects of Δ^9^‐THC are comparable with those obtained at GABAergic synapses in mouse hippocampus in our lab (EC_50_ 1.2 μM, 43 ± 2% maximal inhibition; Laaris *et al*. 2010).

### Inhibition of glutamate release by designer cannabinoids

**Figure 3:**
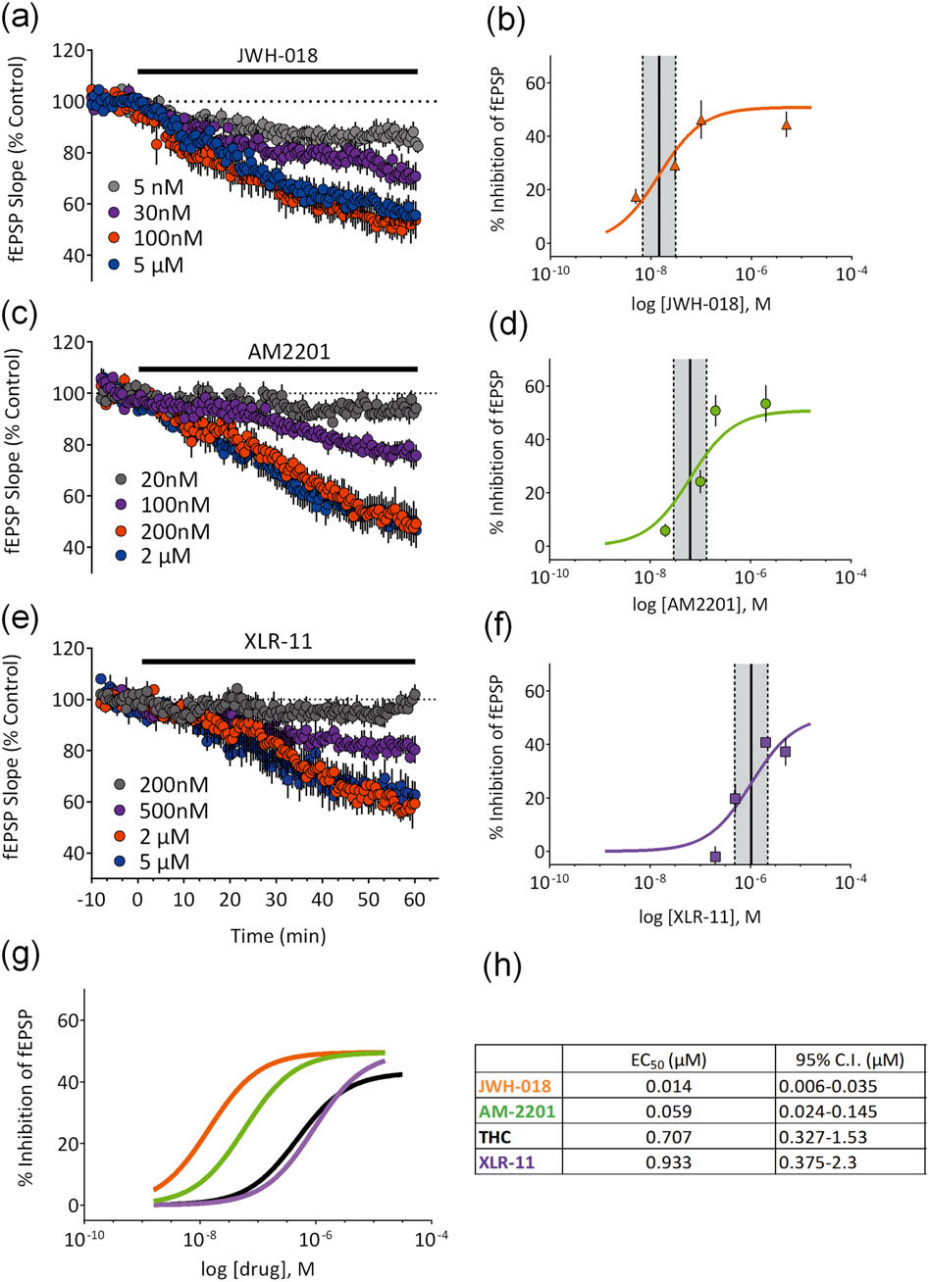
Concentration–response curves at hippocampal CA1 synapses for the Spice constituents JWH‐018, AM2201 and XLR‐11. Left panels (a,c,e) show the time course of each compound at several concentrations; right panels (b,d,f) show concentration–response curves (*n* = 3–8 slices per concentration). The EC_50_ (solid lines) and 95% confidence intervals (dashed lines) are indicated for each compound. (g) shows the curves replotted on the same graph to highlight the potency differences. Note that JWH‐018 (orange trace) is the most potent compound in the series relative to Δ^9^‐tetrahydrocannabinol (THC) (black trace) and that XLR‐11 is similar in potency to Δ^9^‐THC. The table in (h) summarizes the mean calculated EC50 values and 95% confidence intervals for each of the tested compounds. [Colour figure can be viewed at http://wileyonlinelibrary.com]

**Figure 4:**
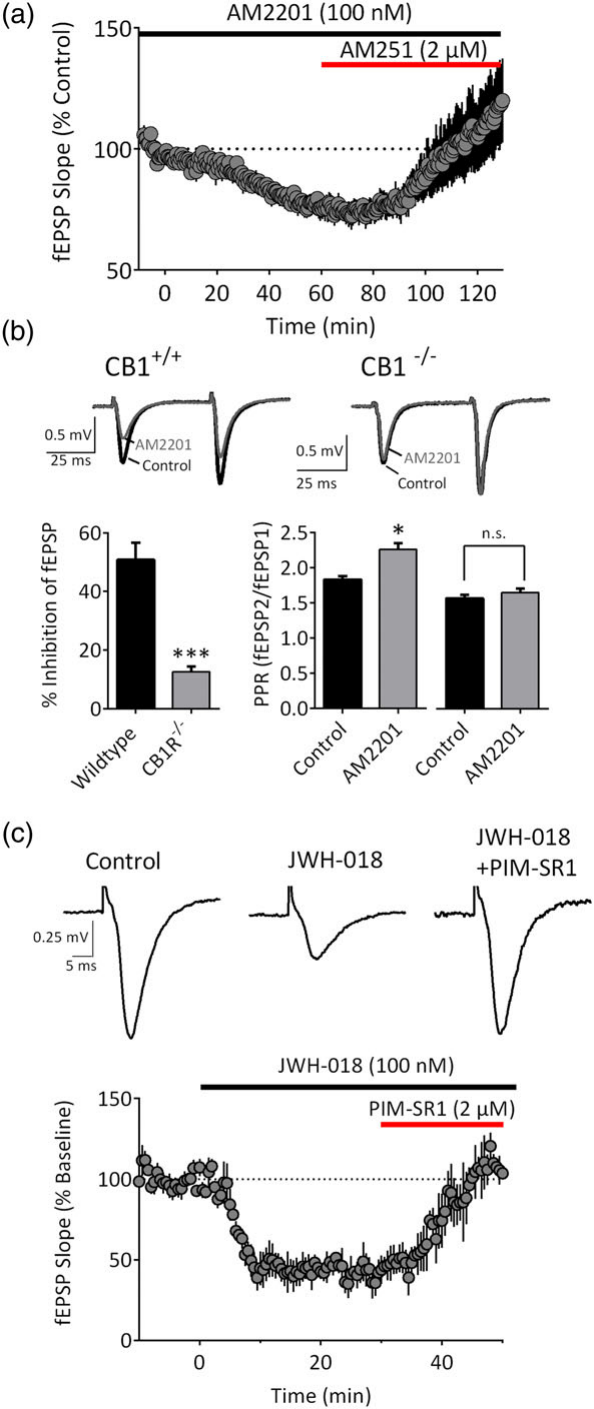
AM2201 and JWH‐018 act via CB1 receptors to inhibit glutamate release. (a) Time course of AM2201 (100 nM) and reversal by the CB1 antagonist/inverse agonist AM251 (*n* = 4). (b) Representative traces demonstrating the effects of AM2201 (200 nM) in slices from a wildtype (CB1+/+) and knockout (CB1−/−) mouse. The summary demonstrates the significant difference (***P < 0.001, *n* = 6, two‐tailed unpaired *t*‐test) in the effects between wildtype and CB1−/− mice. In wildtype mice, AM2201 significantly enhanced the paired‐pulse ratio (*P < 0.05, two‐tailed paired *t*‐test), whereas this effect was absent in CB1−/− mice (*P* = 0.1302, two‐tailed paired *t*‐test). (c) Traces and mean time course (*n* = 3) demonstrating fEPSP inhibition by JWH‐018 (100 nM) and its reversal by PIMSR1 (2 μM). [Colour figure can be viewed at http://wileyonlinelibrary.com]

Among the first generation of synthetic cannabinoid compounds isolated from confiscated synthetic marijuana products was JWH‐018 (Uchiyama *et al*. 2010; Wiley *et al*. 2014b). This compound was previously shown to inhibit glutamate release at hippocampal synapses in culture (Atwood *et al*. 2010). Consistent with these results, JWH‐018 inhibited fEPSPs in mouse brain slices (Fig. 3a), and its EC_50_ (Fig. 3b; 14.0 nM; 95% CI = 6–35 nM) was remarkably similar to that observed in cultured hippocampal neurons (14.9 nM; Atwood *et al*. 2010). The maximal inhibition was 46 ± 7% (*n* = 5) at 100 nM, and this was fully reversed by PIMSR1 (Fig. 4c; *n* = 3, 97 ± 6% of control, *P* = 0.184, two‐tailed paired *t*‐test).

Following the placement of JWH‐018 on the US Drug Enforcement Agency Schedule I list in March 2011 (Drug Enforcement Administration, USA, Department of Justice 2011), a second generation of synthetic cannabinoids began to appear in confiscated products. Among these, a fluoropentyl derivative of JWH‐018 known as AM2201 that binds to the CB1R with low nanomolar affinity (Deng & Makriyannis 2005) was detected in increasing amounts (Fig. 1). We observed that AM2201 also potently inhibited fEPSPs, with an EC_50_ slightly higher than JWH‐018 (Fig. 3c,g; 59 nM; 95% CI = 24–145 nM), and generated a maximal inhibition of 53 ± 7% at 200 nM (*n* = 6; Fig. 3d). Consistent with a presynaptic site of action, AM2201 significantly enhanced the paired pulse ratio in response to paired stimuli (Fig. 4b; *n* = 6, *P* = 0.01, two‐tailed paired *t*‐test). The effect of AM2201 was reversed by bath application of the CB1R antagonist/inverse agonist AM251 (Fig. 4a; *n* = 4, 118 ± 18% of control, *P* = 0.3825, two‐tailed paired *t*‐test), and it had no effect on glutamate release in slices obtained from CB1R knockout mice (Fig. 4b; *n* = 6, *P* < 0.001, *t* = 6.246, d.f. = 10, unpaired *t*‐test versus control).

By the end of the year 2012, a new designer cannabinoid‐like compound was increasingly isolated from confiscated synthetic marijuana products. This synthetic compound was structurally novel and referred to as XLR‐11 [(1‐(5‐fluoropentyl)‐1*H*‐indol‐3‐yl)(2,2,3,3‐tetramethylcyclopropyl)methanone] (Seely *et al*. 2013; U.S. Drug Enforcement Administration Office of Diversion 2013). When the effect of XLR‐11 on glutamatergic fEPSPs was examined, we found that it inhibited these responses with a lower potency than JWH‐018 or AM‐2201 (Fig. 3e,f; EC_50_ = 933 nM; 95% CI = 0.38–2.3 μM). Moreover, XLR‐11 exhibited a trend toward a lower degree of maximal inhibition of fEPSPs compared with these other synthetic cannabinoids (Fig. 3f,g, maximal inhibition = 41 ± 2% at 2 μM; *n* = 4). The inhibition of the response produced by XLR‐11 was also blocked by pre‐treatment of slices with 5 μM PIMSR1 indicating a role for CB1Rs in this response (*n* = 9, 95 ± 3% of control, *P* = 0.02, *t* = 2.348, d.f. = 20 versus 5 μM XLR‐11 alone).

### Disruption of LTP by designer cannabinoids

**Figure 5:**
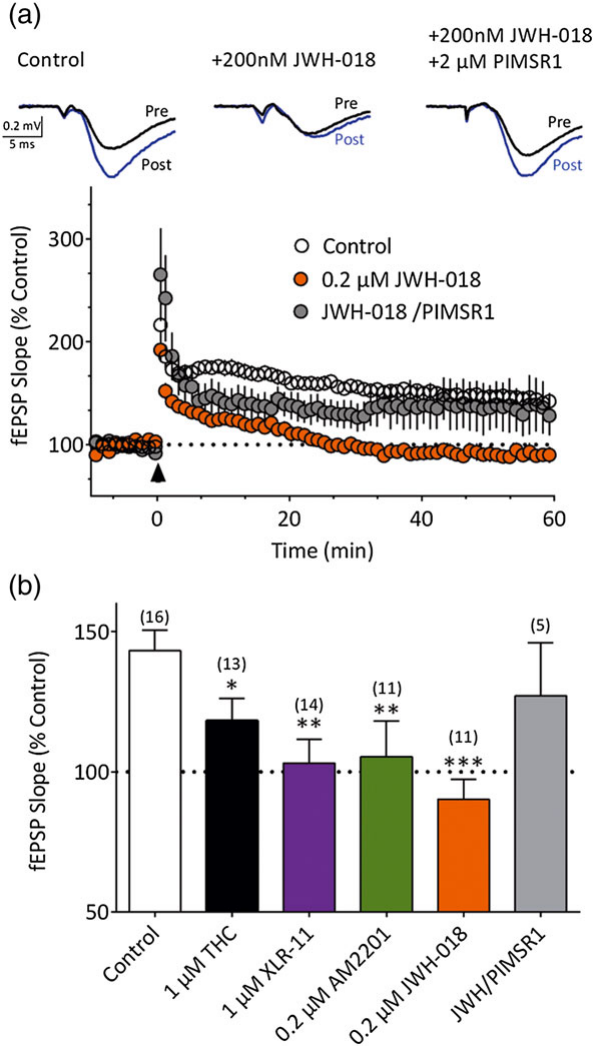
Disruption of hippocampal LTP by synthetic cannabinoids. (a) Representative traces from a control (untreated) slice, a slice pre‐treated for 90 minutes with JWH‐018 (0.2 μM) and a slice pre‐treated with JWH‐018, followed by 2 μM PIMSR1. Traces are averaged from the period immediately prior to (pre) and 60 minutes following (post) high‐frequency stimulation. Time course for all control slices (*n* = 16) and slices pre‐treated with JWH‐018 (*n* = 11) or JWH‐018 with subsequent PIMSR1 application (*n* = 5). LTP was elicited by high‐frequency stimulation delivered at the time indicated by the arrowhead. (b) Average LTP observed in control slices and slices pre‐treated with agonists at the indicated concentrations. The numbers of slices are indicated in parentheses. A one‐way ANOVA detected significant differences (*F*_(5,64)_ = 4.51, *P* = 0.001) among the treatment groups. *P < 0.05, **P < 0.01, ***P < 0.001 versus control; Holm–Sidak multiple comparisons test. [Colour figure can be viewed at http://wileyonlinelibrary.com]

The deleterious effects of cannabinoids on learning and memory are well established in humans (Abel 1970) and in animal models (Wise *et al*. 2009; Han *et al*. 2012). It is hypothesized that the ability of cannabinoid agonists to impair hippocampal long‐term potentiation may play a role in the cognitive impairments produced by these drugs (Misner & Sullivan 1999; Hoffman *et al*. 2007; Abush & Akirav 2010; Basavarajappa & Subbanna 2014; Navakkode & Korte 2014). We therefore evaluated the effects of the synthetic cannabinoids on hippocampal LTP and compared these effects with those of Δ^9^‐THC. In untreated, control slices (*n* = 16), delivery of HFS (3 × 100‐Hz trains) resulted in reliable, stable potentiation of fEPSP slopes (Fig. 5a and b; 143 ± 7% of baseline). In contrast, LTP was absent in slices incubated for 90 minutes in 200 nM JWH‐018 (Fig. 5a and b; *n* = 11, 90 ± 7% of baseline, *P* = 0.0003 versus control). This ability of JWH‐018 to block LTP was prevented in another group of slices when the agonist treatment was followed by application of the CB1R antagonist PIMSR1 (2 μM), beginning 30 minutes prior to HFS (Fig. 5a and b; *n* = 5, 127 ± 19% of baseline, *P* = 0.3320 versus control). As shown in Fig. 5b, slices incubated for 90 minutes with AM‐2201 (200 nM; *n* = 11), XLR‐11 (1 μM; *n* = 14) or Δ^9^‐THC (1 μM; *n* = 13) also showed significantly reduced LTP (AM2201, 105 ± 12% of baseline, *P* = 0.0076 versus control; XLR‐11, 103 ± 8% of baseline, *P* = 0.0035 versus control; Δ^9^‐THC, 118 ± 7% of baseline, *P* = 0.0416 versus control). Together, these results suggest that, in addition to their ability to acutely depress glutamatergic transmission, the synthetic cannabinoids present in ‘Spice’ preparations also limit synaptic plasticity in the hippocampus.

## Discussion

The present study compares the effects of several synthetic cannabinoids recently identified in psychoactive Spice formulations, using a functional synaptic response in a brain area relevant to the cognitive effects of marijuana. Importantly, our studies involved direct comparisons of these synthetic cannabinoids with Δ^9^‐THC, the primary psychoactive active cannabinoid in marijuana. All of the cannabinoid molecules dose‐dependently suppressed hippocampal synaptic glutamate release, with the following relative order of potency: JWH‐018 > AM2201 >> Δ^9^‐THC > XLR‐11, and the effects of all compounds were blocked by the selective CB1R antagonists AM251 or PIMSR1, consistent with the expression of CB1Rs on hippocampal glutamate axon terminals (Kawamura *et al*. 2006). The potency of JWH‐018 is also in agreement with a previous report obtained in cultured hippocampal neurons (Atwood *et al*. 2010). The high potency of JWH‐018 and AM2201 in inhibiting the synaptic response is consistent with the high affinity of these compounds at CB1Rs previously reported in receptor binding assays (*K_i_* = 9 and 1 nM, respectively; Carroll *et al*. 2012), as well as with the ability of low doses of these compounds to substitute for Δ^9^‐THC in drug discrimination assays (Baumann *et al*. 2014; Järbe & Gifford 2014; Wiley *et al*. 2014a). XLR‐11 is structurally distinct from JWH‐018 and AM2201, having been derived from a series of cyclopropylindoles synthesized for potential use as cannabinoid 2 receptor agonists (Frost *et al*. 2010). This compound, which is emerging as a major component of Spice‐related substances (U.S. Drug Enforcement Administration Office of Diversion 2013), has been reported to bind to CB1Rs, with slightly greater affinity than Δ^9^‐THC, and to also substitute for Δ^9^‐THC in a mouse drug discrimination paradigm (Wiley *et al*. 2013). Our results suggest that XLR‐11 is nearly equivalent to Δ^9^‐THC at inhibiting glutamate release via CB1Rs but that both compounds are far less potent than the synthetic constituents found in earlier synthetic marijuana formulations. Based on these observations, it is tempting to speculate that the adverse physiological and psychological effects often associated with use of synthetic marijuana formulated with higher‐affinity CB1R agonists (Winstock & Barratt 2013) may be driving the demand for a synthetic drug with properties closer to those of Δ^9^‐THC. Correspondingly, the incidence of adverse reactions may be smaller with lower‐affinity synthetic cannabinoids.

Despite the differences in potency among the compounds tested, we did not observe statistically significant differences in the maximum inhibition (efficacy) of glutamatergic synaptic transmission. This was surprising for Δ^9^‐THC, because previous studies have suggested that it is a partial agonist at CB1Rs in G‐protein activation assays (Sim *et al*. 1996; Burkey *et al*. 1997) and at inhibition of synaptic responses in cultured hippocampal neurons (Shen & Thayer 1999; Straiker & Mackie 2005). Interestingly, the study by Shen and Thayer reported that Δ^9^‐THC suppressed excitatory transmission by ~60% (Shen & Thayer 1999), which is typically the maximum inhibition seen by cannabinoid agonists *in vitro* and is greater than the effect observed in the present study. We have previously demonstrated that Δ^9^‐THC displays full agonist properties at CB1Rs on hippocampal GABAergic terminals (Laaris *et al*. 2010). However, because tonic adenosine levels limit Δ^9^‐THC effects at hippocampal glutamatergic, but not GABAergic synapses (Hoffman *et al*. 2010), all of the present studies were performed in the presence of the adenosine A1 receptor antagonist DPCPX. It is therefore possible that these conditions permitted us to observe more robust and reliable inhibition of excitatory transmission by Δ^9^‐THC. Together, our results suggest that these compounds primarily differ from Δ^9^‐THC in terms of their relative potency at CB1Rs expressed on hippocampal glutamatergic terminals, rather than in their maximum efficacy.

The ability of synthetic cannabinoids to inhibit neurotransmitter release in the hippocampus likely has consequences for cognitive function. Prior studies have demonstrated that JWH‐018 (Compton *et al*. 2012) and the related naphthoylindole JWH‐081 (Basavarajappa & Subbanna 2014) disrupt spatial learning in rodents. In the present study, all of the drugs tested, including Δ^9^‐THC, suppressed glutamatergic transmission to a similar extent and significantly reduced hippocampal LTP. This is consistent with prior work demonstrating cannabinoid‐mediated inhibition of synaptic plasticity both *in vivo* (Abush & Akirav 2010) and *in vitro* (Basavarajappa & Subbanna 2014; Collins *et al*. 1994; Misner & Sullivan 1999; Navakkode & Korte 2014). In our studies, slices were exposed to each agonist for 90 minutes, followed by evaluation of LTP in agonist‐free aCSF. This was performed in order to standardize the exposure time to the agonists, similar to previous studies (Collins *et al*. 1994, 1995). The slow‐onset time of these agonists to inhibit glutamatergic transmission, coupled with the limited washout of these lipophilic compounds, suggests that they were present in the tissue during the recordings. Consistent with this, PIMSR1 prevented the effect of JWH‐018 on LTP when applied immediately following the 90‐minute pre‐treatment period and 30 minutes prior to HFS. Thus, the most parsimonious explanation of our data is that reduced LTP reflects ongoing suppression of glutamatergic transmission by actions of these compounds at CB1Rs. Indeed, this is the general mechanism through which other CB1 agonists have been demonstrated to block LTP (Collins *et al*. 1994, 1995; Misner & Sullivan 1999; Sullivan 2000). The ability of these and other Spice compounds to disrupt hippocampal LTP is likely to have important consequences for learning and memory (Pastalkova *et al*. 2006; Whitlock *et al*. 2006; Compton *et al*. 2012; Basavarajappa & Subbanna 2014). We have previously demonstrated that repeated exposure to Δ^9^‐THC disrupts hippocampal LTP and alters signaling at both glutamatergic and GABAergic synapses (Hoffman *et al*. 2007). Although we have not yet examined the effects of these compounds on GABAergic axon terminals in the hippocampus, where CB1Rs are also widely expressed (Katona *et al*. 1999; Hoffman & Lupica 2000; Dudok *et al*. 2014), the expected inhibition of GABAergic function likely also contributes to disrupted network activity in the hippocampus, leading to deficits in cognitive function (Hájos *et al*. 2000; Puighermanal *et al*. 2009). In addition, it is possible that the *in vivo* effects of these compounds will reflect the activity of both the parent compound and its metabolites, several of which retain strong biological activity at CB1Rs (Brents *et al*. 2011; Fantegrossi *et al*. 2014). Overall, the present findings demonstrate that synthetic cannabinoids most often found in psychoactive synthetic marijuana products have profound effects on hippocampal neurotransmission and are much more potent than Δ^9^‐THC at activating CB1Rs. This much higher affinity for CB1Rs by synthetic cannabinoids, as compared with Δ^9^‐THC, is likely to prolong the period in which this receptor is activated, thereby extending the duration of behavioral and psychological effects of these drugs. As CB1Rs are ubiquitously expressed in the human brain and extensively engaged by these agonists, the notion that synthetic cannabinoids are benign versions of naturally occurring marijuana is not supported. Given the continued widespread use of these marijuana‐like products, especially by young people, future studies are warranted to elucidate the acute and chronic effects of synthetic cannabinoids on mood, learning and memory, and other physiological processes across development.
